# Development of a group contribution method for estimating free energy of peptides in a dodecane-water system via molecular dynamic simulations

**DOI:** 10.1186/s12859-016-1399-5

**Published:** 2016-12-07

**Authors:** Camilo Andrés Mora Osorio, Andrés Fernando González Barrios

**Affiliations:** Grupo de Diseño de Productos y Procesos (GDPP), Departamento de Ingeniería Química, Universidad de los Andes, Carrera 1E No. 19A-40, Edificio Mario Laserna, Bogotá, DC Colombia

**Keywords:** Molecular dynamics, Free energy calculations, Group contribution method, Residues chain

## Abstract

**Background:**

Calculation of the Gibbs free energy changes of biological molecules at the oil-water interface is commonly performed with Molecular Dynamics simulations (MD). It is a process that could be performed repeatedly in order to find some molecules of high stability in this medium.

Here, an alternative method of calculation has been proposed: a group contribution method (GCM) for peptides based on MD of the twenty classic amino acids to obtain free energy change during the insertion of any peptide chain in water-dodecane interfaces. Multiple MD of the twenty classic amino acids located at the interface of rectangular simulation boxes with a dodecane-water medium were performed.

**Results:**

A GCM to calculate the free energy of entire peptides is then proposed. The method uses the summation of the Gibbs free energy of each amino acid adjusted in function of its presence or absence in the chain as well as its hydrophobic characteristics.

**Conclusion:**

Validation of the equation was performed with twenty-one peptides all simulated using MD in dodecane-water rectangular boxes in previous work, obtaining an average relative error of 16%.

**Electronic supplementary material:**

The online version of this article (doi:10.1186/s12859-016-1399-5) contains supplementary material, which is available to authorized users.

## Background

Many studies of biomolecules have been developed in the last two decades in order to find structures with some exclusive properties that could be interesting in several fields. Biosurfactants are attractive for industry due to their property of lowering the interfacial tension, a property that can be used in different applications to stabilize emulsions such as medicine, food, pharmaceutics, oil spill remediation, personal care, and others [1–4]. The stabilization of these emulsions is given by the minimization of the free energy at the interface. Therefore the calculation of this thermodynamic property is a commonly accepted criterion to evaluate the potential of biomolecules to be biosurfactants [5–7].

Molecular Dynamics (MD) simulations have been used successfully in the last few years to evaluate proteins and peptides as biosurfactants candidates, which have been evaluated in experimental processes with interesting results [7, 8]. Nevertheless, MD to calculate Gibbs free energy change of molecules with peptide size or complex systems composed by different types of biomolecules becomes an inefficient method when fast results are needed [9].

One alternative way to calculate free energy of biomolecules is by using group contribution methods, (GCM). The GCM are based on experimental or in silico data of some specific system, and it can be used to describe the properties of molecules in similar conditions, reducing the time of calculation of thermodynamic properties. Some GCM have been created for the calculation of free energy of proteins, peptides and other molecules and have been tested against results of MD obtained similar results, but they are limited because they are based on experimental procedures [10–12]. Using these methods, the values of free energy of biomolecules are determined by the sum of contributions of each residue that is part of the whole chain assigning a unique energy value to each residue.

The aim of this work is to propose a GCM based on MD for estimating free energy change values of any residues chain without having to resort the simulation of the whole peptide. The GCM is developed using multiple MD of single amino acids and pairs of amino acids located at the interface of dodecane-water simulation boxes. An equation to calculate the value of free energy of an entire peptide relating the presence and absence of a residue as well as its hydrophobic characteristics is proposed. This equation has the capability of calculate the free energy change value during the insertion of peptides at water-oil interfaces. The validation of the equation was performed with twenty-one peptides, and its capability for proteins with *Escherichia coli (E. coli)* OmpA porin which can stabilize dodecane/water mixtures [7, 8].

## Methods

### Gibbs free energy calculations

Gibbs free energy calculations using MD are based on the principle that the free energy is a state function, therefore changes from an A to a B state can be studied only in function of the initial and the final energy states. The change of Gibbs free energy between A and B using MD requires that the Hamiltonian change gradually from one to the other state and this is possible making the Hamiltonian a function of *λ* (coupling parameter) where A corresponds to the state *λ = 0* and B to the state *λ = 1* [13]. Then we have:

1 $$ G\left(\uplambda \right)=-{\mathrm{k}}_{\mathrm{B}}Tln(Q) $$

Where ${\mathrm{k}}_{\mathrm{B}}$ is the Boltzmann constant, $T$ is the temperature and $Q$ is the partition function:

2 $$ Q={\left(N!{h}^{3N}\right)}^{-1}{\displaystyle \iiint exp}\left(-\frac{1}{k_BT}*H\left(p,q;\uplambda \right)\right)dp\ dq\ dV $$

One of the most accepted numerical methods to compute the integral of the derivative of the Hamiltonian over *λ* is the Bennett’s Acceptance Ratio (BAR). In this method the Gibbs free energy from ${\lambda}_i$ to ${\lambda}_j$ is given by [14]:

3 $$ \varDelta {G}^{BAR}\left({\uplambda}_{\mathrm{i}}\to {\uplambda}_{\mathrm{j}}\right)={k}_BT\left(Ln\frac{{\left\langle f\left\langle U\left\langle {\uplambda}_{\mathrm{i}}\right\rangle -U\left\langle {\uplambda}_{\mathrm{j}}\right\rangle +C\right\rangle \right\rangle}_{\uplambda_{\mathrm{j}}}}{{\left\langle f\left\langle U\left\langle {\uplambda}_{\mathrm{j}}\right\rangle -U\left\langle {\uplambda}_{\mathrm{i}}\right\rangle +C\right\rangle \right\rangle}_{\uplambda_{\mathrm{i}}}}\right)+C $$

Where $U$ is the potential energy and,

4 $$ f(x)=\frac{1}{1+ \exp \left(x\left({k}_BT\right)\right)} $$

5 $$ C={k}_BTln\frac{Q_i{N}_j}{Q_j{N}_i} $$

By the iteration of the Eq. 3 until the following condition is fulfilled, the Eq. 3 becomes Eq. 5 [13]:

6 $$ \frac{{\left\langle f\left\langle U\left\langle {\uplambda}_{\mathrm{i}}\right\rangle -U\left\langle {\uplambda}_{\mathrm{j}}\right\rangle +C\right\rangle \right\rangle}_{\uplambda_{\mathrm{j}}}}{{\left\langle f\left\langle U\left\langle {\uplambda}_{\mathrm{j}}\right\rangle -U\left\langle {\uplambda}_{\mathrm{i}}\right\rangle +C\right\rangle \right\rangle}_{\uplambda_{\mathrm{i}}}}=1 $$

7 $$ \varDelta {G}^{BAR}\left({\uplambda}_{\mathrm{i}}\to {\uplambda}_{\mathrm{j}}\right)={k}_BTln\frac{N_j}{N_i}+C $$

The construction of the entire difference of free energy between the states A and B is constructed with the sum of all the possible intermediate states as [13]:

8 $$ \varDelta {G}^{BAR}={\displaystyle \sum_{l=1}^n}\varDelta {G}^{BAR}\left({\uplambda}_{\mathrm{l}}\to {\uplambda}_{\mathrm{l}+1}\right) $$

**Fig. 1:**
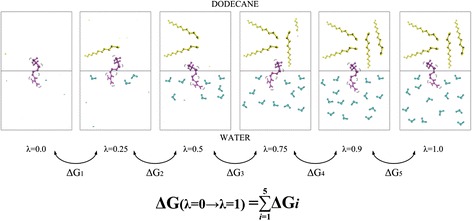
Schematic representation of MD using the coupling parameter λ. The final value of Gibbs free energy change is obtained as the summation of the Gibbs free energy changes between the initial state and the final state using the intermediate *λ-states*. All the simulations were carried out using those six *λ-states*

Where $l=\left(1,\;2,\dots, n\right)$ and ${\uplambda}_1=0,\;{\uplambda}_{\mathrm{n}}=1$ (see Fig. 1). Therefore, *λ-states* selected affect the final free energy value calculated. The greater the number of *λ-states,* the better convergence for free energy calculation; however, it is translated into a greater number of simulations, time of process and computational capability.

### Initial configurations and simulation box construction

**Fig. 2:**
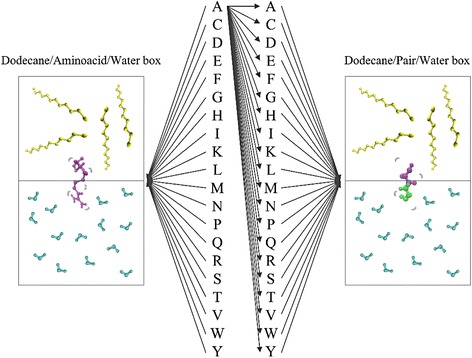
Construction of the forty simulation boxes. The left side of the figure represents the single amino acid box construction where each amino acid was simulated in the interface dodecane-water. The right side of the figure represents the pair in the simulation box, where all the combinations were constructed using Alanine and other amino acid

Two main types of simulations were carried out. Firstly, the twenty amino acids were simulated in a dodecane-water box in order to obtain the individual contribution of each amino acid to the chain. Secondly, twenty pairs were constructed using the program USFC Chimera [15] with the same hydrophobic amino acid as the base (Alanine) and the other nineteen amino acids as the complementary part. These pairs were simulated in a dodecane-water box in order to know the influence of the hydrophobic or hydrophilic characteristics of each couple in the chain (Fig. 2).

**Fig. 3:**
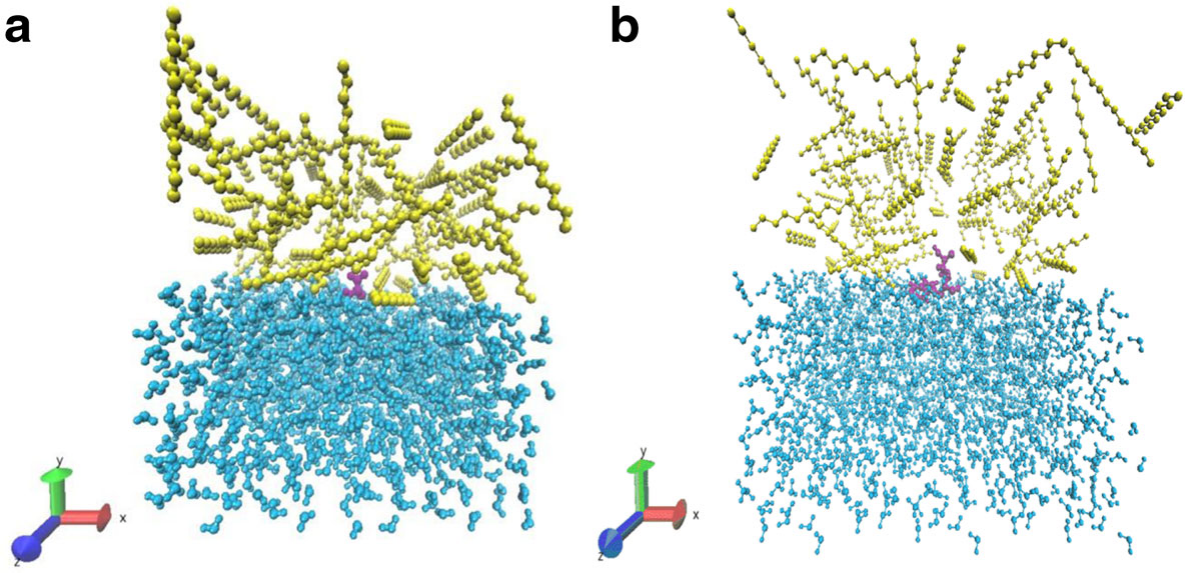
**a-b** Starting configurations of amino acids and pairs of amino acids, the yellow molecules are dodecane, the cyan molecules are water and the purple molecules are one amino acid and one pair of amino acids. In the (**a**) box the amino acid Alanine is placed in the interface while in the (**b**) box the pair Alanine-Tryptophan is placed in the interface

The PDB models of all the amino acids were obtained from the European Molecular Biology Laboratory and European Bioinformatics Institute (EMBL-EBI), which are also found in the RCSB Protein Data Bank [16]. Each amino acid and pair of amino acids was placed in a rectangular simulation box with the box edges at least 2 nm separated from the structure surface. The average dimensions of those amino acid boxes and pair of amino acid boxes were 4.39*4.74*4.31 nanometers and 4.67*4.82*4.40 nanometers respectively (See Additional file 1: Table S1 and S2 in the Electronic supplementary material). The boxes had two compartments, one solvated with dodecane molecules (72 to 92 molecules) and the other solvated with water molecules (1400 to 1650 molecules) in order to simulate the interface. All of the residues and pairs of amino acids were placed in the interface of this configuration using the program USFC Chimera [14] as it is shown in Fig. 3, the visualization program used was Visual Molecular Dynamics (VMD) [17].

### Molecular dynamics simulations

The MD of all the twenty amino acids and twenty pairs were carried out using the GROMACS program, version 4.6.5 [18], the force field used was GROMOS53a6 with the selection of extended simple point water (SPC/E) and dodecane [19]. The energy minimization was developed using the steepest descendent algorithm with a tolerance of 100 KJ*mol^−1^*nm^−1^, a step of 0.01 fs, a Coulomb cut off distance of 1.0 nm, a Van de Waals cut off distance of 0.9 nm and a Fourier spacing of 0.12 nm. The cut off were selected according to average values used in literature by similar MD systems [7, 20–22] and in order to limit the effect of artificial periodicity, using cut off values smaller than half the smallest box length in a rectangular box [23]. 200 ps NVT and NPT equilibrations were performed for each λ-state (Additional file 2: Figure S1A and S1B in the Electronic supplementary material). A 5 ns MD at constant temperature and pressure equilibration was used as starting file for each lambda. Simulations at constant temperature and pressure were carried out with a temperature of 300 K and a pressure of 1 bar, using the Langevin thermostat and the Parrinello-Rahman barostat [19] for the NPT ensemble and the Langevin thermostat for the NVT ensemble [24]. Finally the MD was carried out with a total time of 3 ns using an integration time step of 2 fs and six λ states for each residue.

### Free energy calculations

Six *λ-states* were used as the unphysical states between the physical initial and final states (0 and 1) where 0 represents no interactions at the interface and 1 a full interaction for all cases. The number of states was selected based on a sensitivity analysis method [7]. In order to consider solvent effects on the simulations we carried out MD simulations with the same *λ-states* in pure water and dodecane for arginine and methionine finding no significant statistically differences. Free energy obtained from only two states (0 and 1) may lead to errors and unreliable data, therefore the intermediate states become in a continuous pathway connecting the initial and final states. Free energy values of OmpA in dodecane-water interface were obtained by using seven non-equidistant *λ-states* [22]. However, the size of OmpA is larger than single amino acids and the pairs, therefore the number of *λ*-states evaluated for the forty simulation boxes were lower. Also, due to the size of the amino acids and the pairs of amino acids the simulations were carried out with the Coulomb and van der Waals interactions coupled. The free energy estimates were determinated using the Benett Acceptance Ratio method (BAR) with bisection. Virtual machines with 8-GB RAM with 8 cores per machine and 20 GB of storage were employed. These virtual machines were deployed over UnaCloud opportunistic infrastructure at Universidad de los Andes. MD Simulations took around four hours per *λ-state* and two hundred forty simulations were completed for the forty systems (twenty single amino acids and twenty pairs).

### Construction of the group contribution method equation

The construction of an equation based on the results of the free energy calculations was structured using direct comparison between twenty-one peptides (see Table 1). A previously reported protein with surfactant activity, OmpA was simulated using MD in the same dodecane-water medium in order to evaluate the capability of the equation for predicting free energy changes for proteins [22]. The base of this equation consists in two parts: first, the energy contributions of the presence or absence of each amino acid and second, the hydrophobicity effect on these contributions based on the configuration of pairs of amino acids in the chain of the biomolecule.

**Table 1:** Residue chains of the twenty one peptides used for the free energy comparison [22]

Peptide number	Residue Chain
1	PVVAPAPAPAPEVQTKH
2	QRAALIDCLAPDRRV
3	YQWTNNIGDAHTIG
4	PKDNTWYTGAKLG
5	GKNHDTGVSPVFA
6	MLSLGVSYRFGQG
7	THENQLGAGAFG
8	GAGAFGGYQVNP
9	TRPDNGMLSLGV
10	ALIDCLAPDRR
11	IATRLEYQWTN
12	TGNTCDNVKQR
13	KLGWSQYHDT
14	SVVVLGYTDR
15	QRAALIDCLA
16	DPKDGSVVVL
17	NNNGPTHENQLGAGAFGGYQV
18	IYTRLGGMVWRADTKSNVYGKNHDTGVSPVFAGGV
19	WRADTKSNVYGKNHDTGVSPVFAGGV
20	AHTIGTRPDNGMLSLGVSY
21	VVVLGYTDRIGSDAYNQGLSERRAQSVVDYLI

The hydrophobicity effect was considered under the hypothesis of linear additivity of the residues that comprise the pairs. Therefore, it is assumed that the presence of each residue contributes linearly to the free energy value of the pair as a sum of their individual contributions, and the deviation of this value is a reference of the hydrophobicity influence on the chain’s free energy value. It is expected that pairs composed of the same type of hydrophobicity (a combination of two hydrophobic or two hydrophilic amino acids) obtain the major difference compared to the ideal linear free energy value. Adding one molecule with the same hydrophobicity in the interface increases the tendency of the pair of amioacids to move to one of the two phases, therefore the Gibbs free energy of this configuration must be higher than a pair composed of two residues with different hydrophobicity.

Using the results of the forty simulations (single amino acids and pairs) an equation to calculate the free energy value was proposed, using the results of MD of twenty-one peptides. This equation was constructed using two main concepts. First, the free energy value of one peptide or protein can be calculated as the summation of the free energy value of each amino acid in the biomolecule. Second, the hydrophobicity of each residue affects the total free energy value according to the hydrophobicity interaction between each residue and his neighbor. Finally an equation to calculate the Gibbs free energy of any residues chain biomolecule was obtained.

## Results

### Molecular dynamic simulations and free energy calculations

The calculation of the free energy values was carried out using the BAR tool of the GROMACS simulation package. Using the six λ values chosen, it was possible to obtain a significant representation of the path between the A and B states, where ${\lambda}_1=0$ and ${\lambda}_6=1$. Due to the overlapping of the six histograms in the forty simulations, it is possible to affirm that the pathway between the initial state and the final state is complete; hence, the FE sum of all the *λ-states* represents adequately the total Gibbs free energy of the biomolecule (Additional file 3: Figure S2 and Additional file 4: Figure S3 in the Electronic supplementary material). Finally, the total Gibbs free energy values for all the molecules were obtained (Table 2).

**Table 2:** Free energy contribution of the twenty amino acids and the twenty pairs of amino acids

Amino acid	ΔG [KJ/mol]	+/−	Pair	ΔG [KJ/mol]	+/−
Asp	−388.14	2.87	Ala-Asp	−413.69	4.03
Glu	−356.4	0.82	Ala-Glu	−384.85	2.06
Lys	−294.61	2.34	Ala-Lys	−373.32	2.42
Arg	−270.66	1.31	Ala-Arg	−255.87	1.31
Tyr	−177.3	2.22	Ala-Asn	−235.97	8.07
Trp	−168.78	1.69	Ala-Tyr	−229.8	2.17
Gly	−166.48	0.76	Ala-His	−220.27	3.65
Gln	−166.19	1.15	Ala-Gly	−219.52	1.11
His	−165.46	1.71	Ala-Cys	−218.76	2.7
Ala	−164.27	1.61	Ala-Leu	−218.45	0.77
Asn	−161.24	2.19	Ala-Ala	−218.35	1.49
Leu	−158.99	1.58	Ala-Gln	−217.31	1.47
Val	−157.54	1.74	Ala-Pro	−215.99	0.79
Phe	−157.47	1.18	Ala-Val	−215.68	0.84
Ile	−156.9	1.23	Ala-Ile	−215.08	0.95
Cys	−151.74	1.16	Ala-Ser	−213.13	1.2
Ser	−150.28	0.98	Ala-Phe	−210.77	1.56
Thr	−146.34	0.72	Ala-Trp	−209.8	1.54
Pro	−144.78	1.05	Ala-Met	−207.59	3.6
Met	−138.63	1.58	Ala-Thr	−206.68	1.63

Some amino acids like aspartic acid, glutamic acid, arginine and lysine present higher free energy values. However the histogram shows the same behavior for all the amino acids in the first λ sub-state, with a good overlapping in this specific region. It is possible that the higher free energy values obtained by these amino acids is a consequence of a perturbation in the Hamiltonian calculation due to a sudden change of state. By analyzing the free energy of the first segment of the pathway (λ_1_ = 0 to λ_2_ = 0.25) it is possible to note that the free energy value of these four single amino acids is larger than the others (see Additional file 5: Table S3 and Additional file 6: S4 in the electronic supplementary material).

### Model construction

The construction of the GCM equation was carried out by direct comparison between the free energy values obtained in the MD of twenty-one peptides and the summation of free energy values obtained for all the amino acids contained in the corresponding peptide. Free energy contribution of each residue was added and compared against reported data [12] obtaining that the simulations provide free energy values bigger than expected. Free energy values of single amino acids are around 150 KJ/mol (Table 2). The variations obtained could be due to variables like size, charge and polarity. Then, a group of parameters were needed to correct the equation that calculates the closest free energy values to previous simulated peptides [22].

**Fig. 4:**
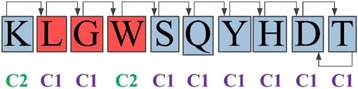
Schematic representation of the residues classification according the hydrophobicity (blue as hydrophilic and red as hydrophobic). The first residue of the peptide is K and it is hydrophilic, the next residue is L and it is hydrophobic, in consequence K is a class 2 residue (C2). The second residue is L and the next residue is hydrophobic too, then L is a class 1 residue (C1). The last residue must be coupled with the previous residue in both directions, in this case D and T are hydrophilic then they are class 1 residues (C1)

The correction parameters were calculated searching the best fit with the in silico available data of twenty-one peptides with a least square approach. Moreover, these parameters were calculated for two specific scenarios of the biomolecule chain configuration. One scenario occurs when the residue evaluated is followed by a residue with the same hydrophobicity i.e., a hydrophobic residue is next to another hydrophobic residue or a hydrophilic residue is next to another hydrophilic residue; this scenario is called *residue class 1* (C1). The other scenario occurs when the residue evaluated is followed by a residue with different hydrophobicity i.e., a hydrophobic residue is next to a hydrophilic residue or a hydrophilic residue is next to a hydrophobic residue; this scenario is called *residue class 2* (C2). The Fig. 4 shows an example using the peptide 13 *(KLGWSQYHDT)*.

The hydrophobicity contribution in the correction parameters was calculated as a deviation percentage between the ideal linear addition of the Gibbs free energy values of the two residues of each pair and the Gibbs free energy values obtained in the MD. Based on the fact that a pair composed by one hydrophobic and one hydrophilic residues must have a lower deviation from the ideal value due to the tail-head effect, the twenty pairs were organized in ascending order to obtain the most representative hydrophobic-hydrophobic pair and the most representative hydrophobic-hydrophilic pair in function of the difference from the ideal additive value (Table 3).

**Table 3:** Deviation of Gibbs free energy values of the twenty pairs of amino acids from the ideal linear value

Pair	Deviation of the free energy value from the ideal linear value [KJ/mol]
Ala-Arg	−179.06
Ala-Asp	−138.72
Ala-Glu	−135.82
Ala-Trp	−123.25
Ala-Gln	−113.15
Ala-Tyr	−111.77
Ala-Gly	−111.23
Ala-Phe	−110.97
Ala-Ala	−110.19
Ala-His	−109.46
Ala-Val	−106.13
Ala-Ile	−106.09
Ala-Leu	−104.81
Ala-Thr	−103.93
Ala-Ser	−101.42
Ala-Cys	−97.25
Ala-Met	−95.31
Ala-Pro	−93.06
Ala-Asn	−89.54
Ala-Lys	−85.56

**Fig. 5:**
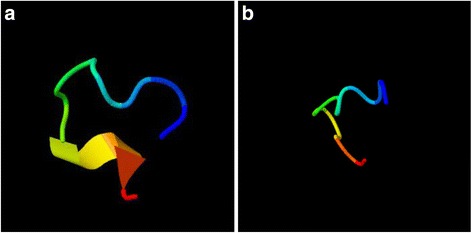
**a-b** Representation of the folding in the two peptides with the greatest error. Peptide 10 (**a**) (ALIDCLAPDRR) and peptide 5 (**b**) (GKNHDTGVSPVFA) show that there is an important folding in the peptide structure which can be associated with the error values reported due the self-interactions of the molecule. Both tertiary structures were obtained using the protein structure predictor I-TASSER [25]

The following pairs: Ala-Arg, Ala-Asp, Ala-Glu, were found to be outliers according to a boxplot diagram, meaning that they provided free energy change values that our model is unable to capture, possibly due to the size of the pair (Table 3). By using the remaining seventeen free energy values, two pairs were selected as the nearest representation of the deviation from the ideality of the hydrophobic-hydrophobic pairs and the hydrophobic-hydrophilic pairs, Ala-Trp and Ala-Lys respectively. The first one has the largest deviation and represents the interactions between two amino acids with the same hydrophobic features. The second one displays the slightest deviation and represents the interactions between two amino acids with different hydrophobic features. Using those representative deviations the α value for the C1 and C2 classifications were adjusted and the final equation of the GCM was proposed as,

9 $$ \begin{array}{l}\varDelta {G}_{molecule}={\displaystyle \sum_{i=A}^Y}{\displaystyle \sum_{j=1}^2}\left(\frac{n\varDelta {G}_{i,\kern0.5em Cj}}{\alpha}\right)\\ {}i=A,C,D,E,F,G,H,I,K,L,M,N,P,Q,R,S,T,V,W,Y\\ {}n= Number\  of\  residues\ C1\  or\ C2\end{array} $$

where $\Delta {G}_{molecule}$ is the Gibbs free of the molecule in KJ/mol, *i* represents each amino acid, C1 or C2 are the class of the residue, *n* is the number of residues class 1 or class 2 according the classification of each one in the molecule and the values of *α* are presented in Table 4. Even though peptides do not frequently fold into typical secondary structures we obtained a deviation effect when comparing GCM equation and the simulation derived values for folded peptides obtained with the protein structure predictor I-TASSER [13] (Fig. 5a and b), then we incorporated a folding correction factor. This factor takes into account the folding potential and was calculated considering the residue position and the turn conformational parameter [4]. A pearson correlation was evaluated for both variables finding a relation between the correlation and the presence of peptide folding (See Additional file 7: Table S5 in in the Electronic supplementary material). Then a potential folding correction was added or subtracted for those peptides with a pearson correlation bigger than 0.40. This correction value was obtained through a linear equation obtained with the pearson correlation and the value needed to equal both free energies obtained (calculated and simulated) when no folding correction is taken into account ($correction\; value=292.8\times Pearson\; correlation-86,6\Big)$.

**Table 4:** Values of *α* according to the amino acid and the class (C1 or C2)

*i*	*j*	α value	*I*	*j*	α value
A	1	8.42	M	1	4.92
A	2	7.69	M	2	4.49
C	1	0.77	N	1	3.85
C	2	0.70	N	2	3.51
D	1	5.35	P	1	9.16
D	2	4.89	P	2	8.36
E	1	5.24	Q	1	1.87
E	2	4.78	Q	2	1.70
F	1	4.66	R	1	8.30
F	2	4.26	R	2	7.57
G	1	8.60	S	1	5.58
G	2	7.84	S	2	5.09
H	1	2.78	T	1	3.57
H	2	2.53	T	2	3.25
I	1	2.87	V	1	8.22
I	2	2.62	V	2	7.50
K	1	4.98	W	1	2.52
K	2	4.54	W	2	2.52
L	1	1.74	Y	1	6.59
L	2	1.59	Y	2	6.59

## Discussion

Gibbs free energy values of twenty-one peptides and the OmpA protein were calculated using the Eq. 9 and these were compared against the reported values [7], (Table 5). The relative error of the twenty-one peptides calculated as,

**Table 5:** Values of the Gibbs free energy for each peptide and the protein OmpA simulated [22] vs calculated with Eq. 9

Peptide	ΔG simulated [KJ/mol]	ΔG calculated [KJ/mol]	Relative Error [%]
1	−489.5	−541.3	10.6
2	−802.8	−889.0	10.7
3	−647.6	−681.9	5.3
4	−589.8	−681.9	15.6
5	−903.2	−454.9	49.6
6	−480.5	−553.0	15.1
7	−554.1	−530.4	4.3
8	−328.2	−360.8	9.9
9	−519.0	−519.6	0.1
10	−517.8	−725.8	40.2
11	−525.9	−601.7	14.4
12	−600.9	−667.1	11.0
13	−501.6	−564.9	12.6
14	−500.3	−372.7	25.5
15	−740.9	−715.0	3.5
16	−604.1	−435.0	28.0
17	−766.7	−863.6	12.6
18	−1295.4	−1282.8	1.0
19	−805.0	−941.9	17.0
20	−607.7	−777.1	27.9
21	−1268.0	−1427.3	12.6
OmpA	−8561.7	−14821.8	73.1

10 $$ \% ErrRel=\frac{\varDelta {G}_{simulated}-\varDelta {G}_{calculated}}{\varDelta {G}_{simulated}}*100 $$

Vary from 0.1 to 33.4% with an average value of 17%. The relative error of the peptides can be explained as a consequence of the self-interaction forces due to folding along the residue chain. Peptide 5 (GKNHDTGVSPVFA) and peptide 10 (ALIDCLAPDRR) present a nonlinear tertiary structure with specific fold sections, obtained using the protein structure predictor I-TASSER [25], which may explain the error values obtained (Fig. 5). Similarly, the free energy value deviation of the remaining peptides can be explained by self-interactions in the folded sections. However the simulation method used with the single amino acids and pairs of amino acids does not provide enough information to reduce the relative error caused by these self-interactions.

Overlap problems in the histograms of the amino acids and pairs of amino acids were analyzed to ensure that the relative error calculated was not dependent of the λ-states. The error associated to each λ-state and the direct inspection of the histograms graphs show that *Trp, Lys, Arg, Asp, Tyr* and *Asn* as single amino acids and *Ala-Arg, Ala-Asn, Ala-Cys, Ala-Gln, Ala-Glu, Ala-His, Ala-Lys, Ala-Met, Ala-Phe*, and *Ala-Tyr* as pairs of amino acids have some smaller overlapping areas than the average, which ultimately affects the free energy values calculation (Additional file 3: Figure S2 and Additional file 4: Figure S3 and Additional file 5: Table S3 and Additional file 6: Table S4 in the in the Electronic supplementary material). However, peptides with the lower relative error calculated, Peptide 9 and Peptide 18 and the higher the relative error calculated, Peptide 5 and peptide 10 confirmed that the overlap problems were not related with the relative error obtained. Then, the overlap problems must be adjusted with extra λ-states at the no overlap areas in order to obtain accurate free energy values, nevertheless these overlap problems play a minor role in the relative error results.

The van der Waals and Coulomb interactions can be evaluated as responsible for the variability of the free energy values obtained. We also evaluated the capability of the equation of predicting free energy change during the insertion in the interface of proteins previously reported with interface activity such as OmpA [8], finding a relative error of 73.1%. Free energy evaluation of OmpA was carried out with a decoupled van der Waals and Coulomb interactions simulation. Then, we believe that van the van der Waals interactions provide higher uncertainties [7] and demonstrated that this GCM equation is more adequate for peptides prediction. The single amino acid and pairs of amino acids simulations were evaluated using a coupled interactions simulation. Hence the uncertainties due to the van der Waals interaction are affecting the final free energy value obtained, nevertheless, it is necessary to modify the simulations uncoupling the interactions in order to know the magnitude of the uncertainty caused by each energetic interaction.

Experimental observations were made upon the addition of some peptides on dodecane-water and crude-water emulsions whose energy was calculated using the GMC based equation. Differential Scanning Calorimetry, droplet size distribution and Interfacial Tension measurements were utilized for the analysis (Manuscript in preparation). Firstly, we found that all sequences displayed a decrease in the interfacial tension corroborating the results predicted by the GMC equation. Secondly we found that water-dodecane emulsions were stable when adding each sequences for more than 7 h indicating that the decrease in surface tension was reflected in emulsion stability. Finally, peptides such as 15, which happen to reach of the lowest free energy values, showed a relation between the surfactant concentration and the crystallization temperatures of n-dodecane and water and took less time to adopt its conformation at the interface.

## Conclusions

A GCM equation was developed to calculate the Gibbs free energy value of biomolecules located in dodecane-water interfaces. MD of the twenty amino acids and twenty pairs were carried out and the free energy values of each one was calculated using the BAR. This equation was constructed based on the minimization of the absolute error between the summation of the free energy results and the free energy values of twenty-one peptides carried out in previous work by our group [7]. Also the GCM equation considers the hydrophobicity of each residue and its contribution to the overall Gibbs free energy value. It was possible using the pairs’ results and the difference of those values from the ideality. Finally, it was proved that the proposed equation can be used as a first approximation with high accuracy to the calculation of Gibbs free energy of short biomolecules.

The interaction of the biomolecule with the environment (not the interface) and the energetic interactions due to the folding become in crucial factors in the overall free energy calculation. Gibbs free energy value obtained to OmpA shows that the length and the folding are two characteristics that must be evaluated in the GCM to improve its accuracy. The relative error is due to the fact that OmpA have specific structural characteristics that could not be taken into account with the simulations performed in this work. Nonetheless, the GCM proposed can be used in future works as a bioprospecting tool in a potential biosurfactants selection in silico workflow.

## Additional files

Additional file 1: Table S1. Size of simulation boxes for single aminoacids. **Table S2.** Size for simulation boxes for pairs simulations. (XLSX 10 kb)
Additional file 2: Figure S1A. Root mean square deviation for the system in Ala-Ala molecular dynamic simulations during the NVT equilibration. **Figure S1B.** Root mean square deviation for the system in Ala-Ala molecular dynamic simulations during the NPT equilibration. (DOCX 91 kb)
Additional file 3: Figure S2. Histograms of the six λ statesof each aminoacid obtained by the use of BAR method. (DOCX 643 kb)
Additional file 4: Figure S3. Histograms of the six states of each dyne obtained by the use of BAR method. (DOCX 1114 kb)
Additional file 5: Table S3. Free energy values of the twenty single aminoacids. (DOCX 554 kb)
Additional file 6: Table S4. Free energy values of twenty dynes. (DOCX 552 kb)

## Abbreviations

BAR: Bennett’s acceptance ratio
GCM: Group contribution method
MD: Molecular dynamics
