# Supplementary material for: Development of a group contribution method for estimating free energy of peptides in a dodecane-water system via molecular dynamic simulations
Source: BMC Bioinformatics. 2016 Dec 7;17:522. doi: 10.1186/s12859-016-1399-5 (PMC5142407; doi:10.1186/s12859-016-1399-5)
Supplement: Additional file 2: Figure S1A. — Root mean square deviation for the system in Ala-Ala molecular dynamic simulations during the NVT equilibration. Figure S1B. Root mean square deviation for the system in Ala-Ala molecular dynamic simulations during the NPT equilibration. (DOCX 91 kb) [file 12859_2016_1399_MOESM2_ESM.docx]

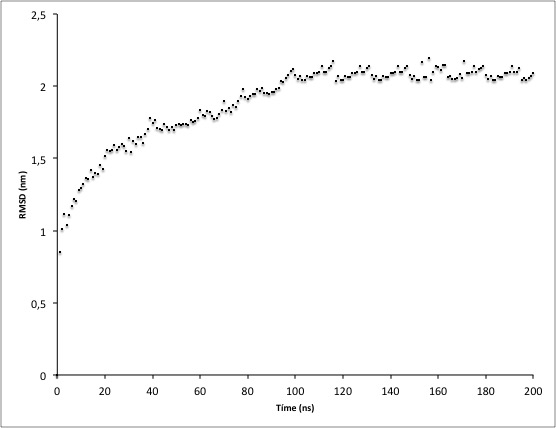


Figure S1A. Root mean square deviation for the system in Ala-Ala molecular dynamic simulations during the NVT equilibration


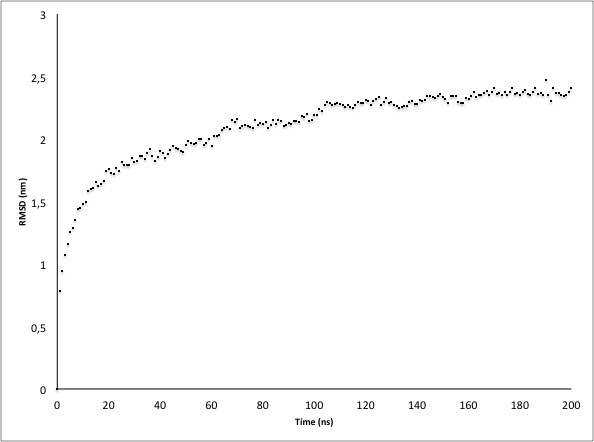


Figure S1B. Root mean square deviation for the system in Ala-Ala molecular dynamic simulations during the NPT equilibration
