# Supplementary material for: Development of a group contribution method for estimating free energy of peptides in a dodecane-water system via molecular dynamic simulations
Source: BMC Bioinformatics. 2016 Dec 7;17:522. doi: 10.1186/s12859-016-1399-5 (PMC5142407; doi:10.1186/s12859-016-1399-5)
Supplement: Additional file 4: Figure S3. — Histograms of the six states of each dyne obtained by the use of BAR method. (DOCX 1114 kb) [file 12859_2016_1399_MOESM4_ESM.docx]

Figure S3. Histograms of the six states of each dyne obtained by the use of BAR method
